# Clinical outcomes of basal insulin and oral antidiabetic agents as an add-on to dual therapy in patients with type 2 diabetes mellitus

**DOI:** 10.1038/s41598-020-62646-z

**Published:** 2020-04-01

**Authors:** Chih-Ning Cheng, Chih-Yuan Wang, Hung-Wei Lin, Ting-Yu Chang, Hsu-Ju Lin, Chiahung Chou, Fang-Ju Lin

**Affiliations:** 1 Graduate Institute of Clinical Pharmacy, College of Medicine, National Taiwan University, Taipei, Taiwan; 2 Department of Internal Medicine, National Taiwan University Hospital, Taipei, Taiwan; 3 School of Pharmacy, College of Medicine, National Taiwan University, Taipei, Taiwan; 4 Department of Health Outcomes Research and Policy, Harrison School of Pharmacy, Auburn University, Auburn, Alabama USA; 5 Department of Medical Research, China Medical University Hospital, Taichung, Taiwan; 6 Department of Pharmacy, National Taiwan University Hospital, Taipei, Taiwan

**Keywords:** Outcomes research, Endocrine system and metabolic diseases

## Abstract

While basal insulin remains the most effective antidiabetic agent and substantially reduces the risk of hypoglycemia, few studies have examined the comparative effect of basal insulin in the real-world setting. This study aimed to assess the outcomes of adding basal insulin compared with thiazolidinediones (TZDs) or dipeptidyl peptidase-4 inhibitors (DPP-4is) as a third antidiabetic agent in patients with type 2 diabetes mellitus (T2DM). A retrospective cohort study involving T2DM was conducted with health administrative data in Taiwan. Patients starting a third antidiabetic agent after receiving a metformin-containing dual combination were identified. The study endpoints included composite major adverse cardiovascular events (MACEs), all-cause mortality, and hypoglycemia. Propensity score matching and Cox modeling were used for analysis. After matching, the basal insulin and TZD groups contained 6,101 and 11,823 patients, respectively, and the basal insulin and DPP-4i groups contained 6,051 and 11,900 patients, respectively. TZDs and DPP-4is were both associated with similar risks of MACEs and hypoglycemia but a lower risk of all-cause mortality than basal insulin (TZDs: HR 0.55, 95% CI 0.38–0.81; DPP-4is: HR 0.56, 95% CI 0.39–0.82). Further studies are needed to elucidate the findings of increased all-cause mortality risk in patients receiving basal insulin, especially those with advanced diabetes.

## Introduction

Diabetes mellitus (DM) substantially increases the risk of cardiovascular diseases (CVDs) and associated deaths; therefore, intensive glucose control has long been considered a gold standard to reduce the occurrence of CVDs^1^. The 10-year follow-up study of the UKPDS (UK Prospective Diabetes Study) revealed a beneficial long-term effect of early intensive glucose control on the reduction in macrovascular events^2^, although this effect did not appear in other clinical trials with advanced DM populations and relatively short follow-up periods^3–5^. Insulin is regarded as the most effective antidiabetic agent for glycemic control, and it possesses a better ability in the preservation of β-cell function than oral hypoglycemic agents (OHAs)^6–8^. The emergence of long-acting basal insulin ameliorates the problem of insulin-associated hypoglycemia^9^. However, the initiation of insulin therapy is often delayed in clinical practice because most patients are reluctant to or inconvenienced by using injectable medications^10^.

The role of basal insulin as an add-on antidiabetic agent remains unclear^11–13^. Previous studies have shown that the combination of metformin and sulfonylureas dominated in dual therapy in the early phase of disease management^14–17^, and other OHAs, such as thiazolidinediones (TZDs) and dipeptidyl peptidase-4 inhibitors (DPP-4is), are common treatment options after the failure of dual therapy^11,13,16^. Several observational studies compared insulin to OHAs and noted a significantly increased risk of CVDs or all-cause mortality associated with insulin^18–23^. However, most of these studies included short-acting insulin in their analysis, and the related hypoglycemic events might have increased the calculated risk of CVDs^18,20–22^. Only few studies, including the ORIGIN (Outcome Reduction with an Initial Glargine Intervention) trial, have specifically examined the risk of cardiovascular events linked to basal insulin^19,23,24^. Therefore, this study aimed to investigate the risk of cardiovascular events, all-cause mortality, and severe hypoglycemic events associated with basal insulin compared with TZDs or DPP-4is as an add-on antidiabetic agent following dual OHA combination therapy.

## Results

### Patient characteristics

**Figure 1:**
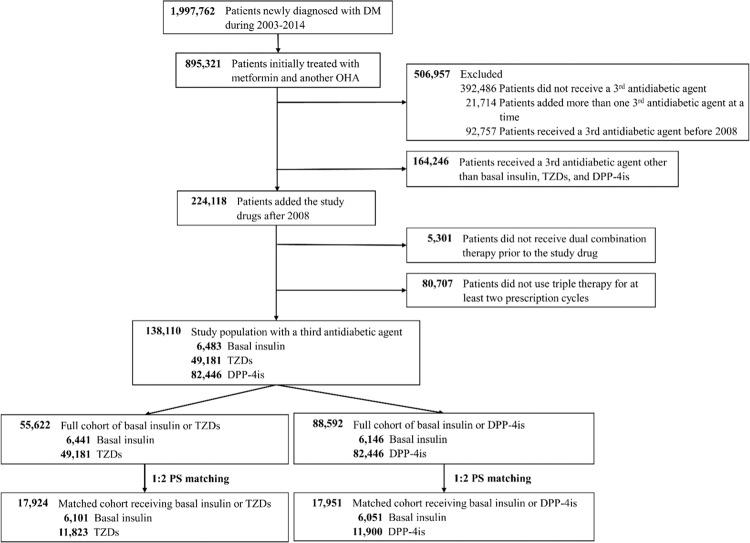
Flow chart of study population selection. Abbreviations: DM, diabetes mellitus; DPP-4is, dipeptidylpeptidase-4 inhibitors; OHAs, oral hypoglycemic agents; TZDs, thiazolidinediones.

Of the 1,997,762 adult patients diagnosed with new-onset DM between 2003 and 2014, 138,110 patients who were administered a third antidiabetic agent were included in the analysis (Fig. 1). There were 6,483 patients (4.7%) in the basal insulin group, 49,181 patients (35.6%) in the TZD group, and 82,446 patients (59.7%) in the DPP-4i group. The basal insulin group contained approximately 10%, 56%, and 34% of patients with NPH, insulin glargine, and insulin detemir, respectively. In each treatment group, approximately 89% of the patients received dual therapy consisting of metformin and sulfonylureas before receiving an intensification therapy agent. In the basal insulin group, previous dual therapy consisting of TZDs and DPP-4is was administered in 42 and 337 patients, respectively, and these patients were excluded from the following analyses of their respective drug classes. Figure 1 shows the process of study population selection and the final cohorts included in the outcome analysis after 1:2 propensity score (PS) matching.

Tables 1 and 2 present the cohort characteristics before and after PS matching. Before matching, the patients in the basal insulin group were younger, were male-dominated, were more likely to receive dual therapy as the initial treatment, had shorter DM duration, had lower HbA1c order rates in one year, and had more hospitalizations and emergency room (ER) visits than those in the TZD and DPP-4i groups. In addition, patients in the basal insulin group had lower prevalence rates of hypertension and dyslipidemia and used less related drugs, including angiotensin-converting enzyme (ACE) inhibitors/angiotensin receptor blockers (ARBs), calcium channel blockers (CCBs), and statins, than those in the other groups. After PS matching, no significant differences in patient characteristics were found between the treatment groups. The median daily dose of TZDs and DPP-4is were 0.7 and 0.9 defined daily dose (DDD)^25^, respectively.

**Table 1:** Baseline characteristics of the patients who received basal insulin or a TZD as a third antidiabetic agent.

Characteristics	Full cohort before matching			Propensity score-matched (1:2) cohort		
	Basal insulin (n = 6,441)	TZDs (n = 49,181)	S.D.	Basal insulin (n = 6,101)	TZDs (n = 11,823)	S.D.
Age, mean ± SD	52.6 ± 12.6	55.8 ± 10.8	0.28	52.9 ± 12.5	53.8 ± 11.6	0.08
Male, n (%)	4,087 (63.5)	27,745 (56.4)	0.15	3,822 (62.7)	7,378 (62.4)	0.01
Index year, n (%)			0.23			0.04
2008–2009	1,641 (25.5)	15,987 (32.5)		1,613 (26.4)	3,269 (27.7)	
2010–2011	1,890 (29.3)	16,133 (32.8)		1,837 (30.1)	3,650 (30.9)	
2012–2013	1,924 (29.9)	11,968 (24.3)		1,768 (29.0)	3,302 (27.9)	
2014	986 (15.3)	5,093 (10.4)		883 (14.5)	1,602 (13.6)	
First DM drug, n (%)			0.34			0.05
Metformin	1,076 (16.7)	9,874 (20.1)		1,066 (17.5)	2,036 (17.2)	
Other OHA	1,279 (19.9)	15,986 (32.5)		1,271 (20.8)	2,688 (22.7)	
Dual therapy as the initial treatment	4,086 (63.4)	23,321 (47.4)		3,764 (61.7)	7,099 (60.0)	
Type of OHA used with metformin for dual therapy			0.28			0.05
Sulfonylureas	5,826 (90.5)	47,602 (96.8)		5,635 (92.4)	11,069 (93.6)	
DPP-4is	337 (5.2)	649 (1.3)		251 (4.1)	395 (3.3)	
Meglitinides	249 (3.9)	650 (1.3)		187 (3.1)	305 (2.6)	
α-glucosidase inhibitors	29 (0.5)	280 (0.6)		28 (0.5)	54 (0.5)	
Time since the first DM drug (years), mean ± SD	2.6 ± 2.8	3.7 ± 2.5	0.43	2.7 ± 2.8	2.9 ± 2.5	0.07
Time since the second DM drug (years), mean ± SD	2.0 ± 2.4	2.9 ± 2.3	0.39	2.1 ± 2.4	2.2 ± 2.2	0.07
aDCSI			0.16			0.03
0	3,119 (48.4)	24,061 (48.9)		2,959 (48.5)	5,779 (48.9)	
1	1,247 (19.4)	12,231 (24.9)		1,223 (20.1)	2,489 (21.1)	
≥2	2,075 (32.2)	12,889 (26.2)		1,919 (31.5)	3,555 (30.1)	
HbA1c order count in 1 year			0.38			0.06
≤2	3,790 (58.8)	19,955 (40.6)		3,465 (56.8)	6,359 (53.8)	
3–4	1,790 (27.8)	20,927 (42.6)		1,778 (29.1)	3,711 (31.4)	
5–6	655 (10.2)	6,731 (13.7)		653 (10.7)	1,360 (11.5)	
>6	206 (3.2)	1,568 (3.2)		205 (3.4)	393 (3.3)	
Baseline PDC of dual therapy (%)			0.26			0.05
≤30	1,048 (16.3)	6,003 (12.2)		1,024 (16.8)	1,985 (16.8)	
30–50	838 (13.0)	9,522 (19.4)		837 (13.7)	1,756 (14.9)	
50–80	999 (15.5)	10,508 (21.4)		993 (16.3)	2,077 (17.6)	
>80	3,556 (55.2)	23,148 (47.1)		3,247 (53.2)	6,005 (50.8)	
Hypoglycemia history, n (%)	52 (0.8)	127 (0.3)	0.08	44 (0.7)	72 (0.6)	0.01
Number of outpatients visits in 1 year			0.37			0.03
≤12	2,087 (32.4)	8,449 (17.2)		1,827 (30.0)	3,406 (28.8)	
13–24	2,017 (31.3)	20,525 (41.7)		1,984 (32.5)	3,898 (33.0)	
25–36	1,176 (18.3)	11,219 (22.8)		1,154 (18.9)	2,316 (19.6)	
37–48	581 (9.0)	4,708 (9.6)		569 (9.3)	1,114 (9.4)	
>48	580 (9.0)	4,280 (8.7)		567 (9.3)	1,089 (9.2)	
Number of hospitalizations in 1 year			0.32			0.03
0	1,483 (23.0)	17,439 (35.5)		1,472 (24.1)	2,981 (25.2)	
1–2	2,359 (36.6)	18,105 (36.8)		2,208 (36.2)	4,191 (35.5)	
≥3	2,599 (40.4)	13,637 (27.7)		2,421 (39.7)	4,651 (39.3)	
Number of ER admissions in 1 year			0.40			0.04
0	4,036 (62.7)	39,481 (80.3)		3,973 (65.1)	7,918 (67.0)	
1	1,512 (23.5)	6,765 (13.8)		1,340 (22.0)	2,463 (20.8)	
≥2	893 (13.9)	2,935 (6.0)		788 (12.9)	1,442 (12.2)	
Cardiovascular history, n (%)	1,929 (30.0)	16,516 (33.6)	0.08	1,891 (31.0)	3,710 (31.4)	0.01
Comorbidities, n (%)						
Myocardial infarction	115 (1.8)	755 (1.5)	0.02	112 (1.8)	204 (1.7)	0.01
Other coronary artery disease	1,021 (15.9)	9,700 (19.7)	0.10	1,011 (16.6)	1,981 (16.8)	0.00
Cerebrovascular disease	735 (11.4)	4,909 (10.0)	0.05	712 (11.7)	1,329 (11.2)	0.01
Hypertension	3,410 (52.9)	31,667 (64.4)	0.23	3,338 (54.7)	6,532 (55.3)	0.01
Dyslipidemia	3,756 (58.3)	35,373 (71.9)	0.29	3,667 (60.1)	7,383 (62.5)	0.05
Heart failure	392 (6.1)	2,845 (5.8)	0.01	385 (6.3)	706 (6.0)	0.01
Peripheral vascular disease	227 (3.5)	2,115 (4.3)	0.04	226 (3.7)	402 (3.4)	0.02
Dysrhythmia	402 (6.2)	3,542 (7.2)	0.04	389 (6.4)	757 (6.4)	0.00
Valvular heart disease	178 (2.8)	1,299 (2.6)	0.01	174 (2.9)	319 (2.7)	0.01
Depression	301 (4.7)	1,406 (2.9)	0.10	276 (4.5)	518 (4.4)	0.01
Bipolar disorder	107 (1.7)	443 (0.9)	0.07	97 (1.6)	174 (1.5)	0.01
Schizophrenia	118 (1.8)	570 (1.2)	0.06	113 (1.9)	226 (1.9)	0.00
Anxiety	1,208 (18.8)	9,160 (18.6)	0.00	1,143 (18.7)	2,259 (19.1)	0.01
Chronic kidney disease	126 (2.0)	1,011 (2.1)	0.01	123 (2.0)	266 (2.3)	0.02
Malignancy	469 (7.3)	2,330 (4.7)	0.11	437 (7.2)	821 (6.9)	0.01
Autoimmune disease	868 (13.5)	5,841 (11.9)	0.05	835 (13.7)	1,594 (13.5)	0.01
Transplantation	12 (0.2)	42 (0.1)	0.03	12 (0.2)	21 (0.2)	0.00
Asthma/COPD	1,108 (17.2)	8,416 (17.1)	0.00	1,069 (17.5)	2,005 (17.0)	0.01
Comedications, n (%)						
α-blockers	190 (3.0)	1,425 (2.9)	0.00	182 (3.0)	340 (2.9)	0.01
β-blockers	1,565 (24.3)	13,345 (27.1)	0.06	1,517 (24.9)	2,944 (24.9)	0.00
ACEIs/ARBs	2,574 (40.0)	23,864 (48.5)	0.17	2,514 (41.2)	4,964 (42.0)	0.02
Calcium channel blockers	2,205 (34.2)	20,232 (41.1)	0.14	2,141 (35.1)	4,183 (35.4)	0.01
Diuretics	1,176 (18.3)	7,798 (15.9)	0.06	1,103 (18.1)	2,062 (17.4)	0.02
Renin inhibitors	9 (0.1)	86 (0.2)	0.01	9 (0.2)	20 (0.2)	0.01
Statins	2,428 (37.7)	23,664 (48.1)	0.21	2,375 (38.9)	4,824 (40.8)	0.04
Fibrates	1,103 (17.1)	10,652 (21.7)	0.11	1,076 (17.6)	2,184 (18.5)	0.02
Other lipid-lowering agents	189 (2.9)	1,172 (2.4)	0.03	178 (2.9)	345 (2.9)	0.00
Aspirin	1,521 (23.6)	12,416 (25.3)	0.04	1,471 (24.1)	2,878 (24.3)	0.01
P2Y12 inhibitors	173 (2.7)	1,000 (2.0)	0.04	170 (2.8)	287 (2.4)	0.02
Other antiplatelets	359 (5.6)	3,273 (6.7)	0.05	351 (5.8)	715 (6.1)	0.01
Anticoagulants	97 (1.5)	360 (0.7)	0.07	88 (1.4)	154 (1.3)	0.01
Nitrates	472 (7.3)	3,146 (6.4)	0.04	452 (7.4)	871 (7.4)	0.00
Digoxin	129 (2.0)	598 (1.2)	0.06	125 (2.1)	212 (1.8)	0.02
Atypical antipsychotics	385 (6.0)	1,788 (3.6)	0.11	357 (5.9)	658 (5.6)	0.01
Systemic steroids	1,701 (26.4)	11,443 (23.3)	0.07	1,600 (26.2)	3,041 (26.7)	0.01

Abbreviations: ACEIs, angiotensin-converting enzyme inhibitors; aDCSI, adapted diabetes complication severity index; ARBs, angiotensin receptor blockers; COPD, chronic obstructive pulmonary disease; DM, diabetes mellitus; DPP-4is, dipeptidylpeptidase-4 inhibitors; ER, emergency room; OHA, oral hypoglycemic agent; PDC, proportion of days covered; S.D., standardized difference; SUs, sulfonylureas; TZD, thiazolidinedione.

**Table 2:** Baseline characteristics of the patients who received basal insulin or a DPP-4i as a third anti-diabetic agent.

Characteristics	Full cohort before matching			Propensity score-matched (1:2) cohort		
	Basal insulin (n = 6,146)	DPP-4is (n = 82,446)	S.D.	Basal insulin (n = 6,051)	DPP-4is (n = 11,900)	S.D.
Age, mean ± SD	52.6 ± 12.5	56.7 ± 11.5	0.34	52.7 ± 12.5	53.2 ± 11.8	0.04
Male, n (%)	3,899 (63.4)	47,271 (57.3)	0.13	3,828 (63.3)	7,512 (63.1)	0.00
Index year, n (%)			0.71			0.04
2008–2009	1,645 (26.8)	4,477 (5.4)		1,551 (25.6)	2,845 (23.9)	
2010–2011	1,862 (30.3)	19,110 (23.2)		1,861 (30.8)	3,707 (31.2)	
2012–2013	1,795 (29.2)	40,105 (48.6)		1,795 (29.7)	3,620 (30.4)	
2014	844 (13.7)	18,754 (22.8)		844 (14.0)	1,728 (14.5)	
First DM drug, n (%)			0.32			0.03
Metformin	1,012 (16.5)	21,711 (26.3)		1,009 (16.7)	2,108 (17.7)	
Other OHA	1,276 (20.8)	21,716 (26.3)		1,266 (20.9)	2,504 (21.0)	
Dual therapy as the initial treatment	3,858 (62.8)	39,019 (47.3)		3,776 (62.4)	7,288 (61.2)	
Type of OHA used with metformin for dual therapy			0.17			0.03
Sulfonylureas	5,826 (94.8)	78,989 (95.8)		5,744 (94.9)	11,337 (95.3)	
TZDs	42 (0.7)	791 (1.0)		42 (0.7)	89 (0.8)	
Meglitinides	249 (4.1)	1,469 (1.8)		236 (3.9)	409 (3.4)	
α-glucosidase inhibitors	29 (0.5)	1,197 (1.5)		29 (0.5)	65 (0.6)	
Time since the first DM drug (years), mean ± SD	2.7 ± 2.8	3.9 ± 3.0	0.44	2.7 ± 2.8	2.7 ± 2.7	0.02
Time since the second DM drug (years), mean ± SD	2.1 ± 2.4	2.9 ± 2.7	0.35	2.1 ± 2.4	2.1 ± 2.3	0.02
aDCSI			0.11			0.04
0	2,945 (47.9)	35,520 (43.1)		2,907 (48.0)	5,881 (49.4)	
1	1,206 (19.6)	19,327 (23.4)		1,197 (19.8)	2,403 (20.2)	
≥2	1,995 (32.5)	27,599 (33.5)		1,947 (32.2)	3,616 (30.4)	
HbA1c order count in 1 year			0.44			0.03
≤2	3,546 (57.7)	30,534 (37.0)		3,455 (57.1)	6,634 (55.8)	
3–4	1,757 (28.6)	31,279 (37.9)		1,754 (29.0)	3,535 (29.7)	
5–6	643 (10.5)	15,784 (19.1)		642 (10.6)	1,345 (11.3)	
>6	200 (3.3)	4,849 (5.9)		200 (3.3)	386 (3.2)	
Baseline PDC of dual therapy (%)			0.28			0.01
≤30	1,023 (16.6)	9,799 (11.9)		1,007 (16.6)	1,982 (16.7)	
30–50	822 (13.4)	19,051 (23.1)		819 (13.5)	1,654 (13.9)	
50–80	983 (16.0)	14,718 (17.9)		981 (16.2)	1,955 (16.4)	
>80	3,318 (54.0)	38,878 (47.2)		3,242 (53.6)	6,309 (53.0)	
Hypoglycemia history, n (%)	49 (0.8)	438 (0.5)	0.03	47 (0.8)	86 (0.7)	0.01
Number of outpatients visits in 1 year			0.29			0.02
≤12	1,940 (31.6)	15,815 (19.2)		1,883 (31.1)	3,689 (31.0)	
13–24	1,946 (31.7)	31,984 (38.8)		1,932 (31.9)	3,885 (32.7)	
25–36	1,131 (18.4)	18,688 (22.7)		1,120 (18.5)	2,157 (18.1)	
37–48	567 (9.2)	8,371 (10.2)		563 (9.3)	1,134 (9.5)	
>48	562 (9.1)	7,588 (9.2)		553 (9.1)	1,035 (8.7)	
Number of hospitalizations in 1 year			0.28			0.04
0	1,405 (22.9)	27,139 (32.9)		1,404 (23.2)	2,893 (24.3)	
1–2	2,233 (36.3)	31,258 (37.9)		2,211 (36.5)	4,453 (37.4)	
≥3	2,508 (40.8)	24,049 (29.2)		2,436 (40.3)	4,554 (38.3)	
Number of ER admissions in 1 year			0.26			0.02
0	3,875 (63.1)	61,688 (74.8)		3,853 (63.7)	7,677 (64.5)	
1	1,432 (23.3)	13,953 (16.9)		1,381 (22.8)	2,705 (22.7)	
≥2	839 (13.7)	6,805 (8.3)		817 (13.5)	1,518 (12.8)	
Cardiovascular history, n (%)	1,862 (30.3)	34,017 (41.3)	0.23	1,850 (30.6)	3,669 (30.8)	0.01
Comorbidities, n (%)						
Myocardial infarction	107 (1.7)	2,994 (3.6)	0.12	107 (1.8)	218 (1.8)	0.00
Other coronary artery disease	991 (16.1)	20,207 (24.5)	0.21	985 (16.3)	1,918 (16.1)	0.00
Cerebrovascular disease	707 (11.5)	11,851 (14.4)	0.09	702 (11.6)	1,274 (10.7)	0.03
Hypertension	3,302 (53.7)	54,774 (66.4)	0.26	3,270 (54.0)	6,425 (54.0)	0.00
Dyslipidemia	3,623 (59.0)	58,217 (70.6)	0.25	3,589 (59.3)	7,170 (60.3)	0.02
Heart failure	377 (6.1)	6,883 (8.4)	0.09	377 (6.2)	706 (5.9)	0.01
Peripheral vascular disease	221 (3.6)	3,455 (4.2)	0.03	220 (3.6)	433 (3.6)	0.00
Dysrhythmia	389 (6.3)	7,954 (9.7)	0.12	387 (6.4)	732 (6.2)	0.01
Valvular heart disease	175 (2.9)	3,560 (4.3)	0.08	174 (2.9)	331 (2.8)	0.01
Depression	291 (4.7)	3,030 (3.7)	0.05	285 (4.7)	514 (4.3)	0.02
Bipolar disorder	98 (1.6)	854 (1.0)	0.05	94 (1.6)	145 (1.2)	0.03
Schizophrenia	115 (1.9)	902 (1.1)	0.06	112 (1.9)	190 (1.6)	0.02
Anxiety	1,163 (18.9)	16,745 (20.3)	0.03	1,144 (18.9)	2,216 (18.6)	0.01
Chronic kidney disease	122 (2.0)	2,235 (2.7)	0.05	122 (2.0)	238 (2.0)	0.00
Malignancy	444 (7.2)	5,401 (6.6)	0.03	440 (7.3)	817 (6.9)	0.02
Autoimmune disease	835 (13.6)	11,279 (13.7)	0.00	824 (13.6)	1,597 (13.4)	0.01
Transplantation	12 (0.2)	133 (0.2)	0.01	12 (0.2)	23 (0.2)	0.00
Asthma/COPD	1,056 (17.2)	15,755 (19.1)	0.05	1,047 (17.3)	2,004 (16.8)	0.01
Comedications, n (%)						
α-blockers	187 (3.0)	3,112 (3.8)	0.04	186 (3.1)	365 (3.1)	0.00
β-blockers	1,505 (24.5)	25,780 (31.3)	0.15	1,489 (24.6)	2,840 (23.9)	0.02
ACEIs/ARBs	2,475 (40.3)	44,391 (53.8)	0.27	2,459 (40.6)	4,846 (40.7)	0.00
Calcium channel blockers	2,121 (34.5)	36,781 (44.6)	0.21	2,103 (34.8)	4,109 (34.5)	0.00
Diuretics	1,131 (18.4)	13,529 (16.4)	0.05	1,104 (18.2)	2,093 (17.6)	0.02
Renin inhibitors	9 (0.2)	470 (0.6)	0.07	9 (0.2)	20 (0.2)	0.00
Statins	2,324 (37.8)	42,554 (51.6)	0.28	2,311 (38.2)	4,655 (39.1)	0.02
Fibrates	1,073 (17.5)	14,870 (18.0)	0.02	1,055 (17.4)	2,101 (17.7)	0.01
Other lipid-lowering agents	182 (3.0)	4,141 (5.0)	0.11	182 (3.0)	395 (3.3)	0.02
Aspirin	1,462 (23.8)	24,651 (29.9)	0.14	1,448 (23.9)	2,779 (23.4)	0.01
P2Y12 inhibitors	163 (2.7)	3,900 (4.7)	0.11	163 (2.7)	326 (2.7)	0.00
Other antiplatelets	348 (5.7)	5,030 (6.1)	0.02	344 (5.7)	681 (5.7)	0.00
Anticoagulants	90 (1.5)	1,501 (1.8)	0.03	90 (1.5)	170 (1.4)	0.00
Nitrates	449 (7.3)	8,838 (10.7)	0.12	445 (7.4)	868 (7.3)	0.00
Digoxin	125 (2.0)	1,546 (1.9)	0.01	124 (2.1)	235 (2.0)	0.01
Atypical antipsychotics	372 (6.1)	3,239 (3.9)	0.10	366 (6.1)	661 (5.6)	0.02
Systemic steroids	1,628 (26.5)	20,199 (24.5)	0.05	1,600 (26.4)	3,143 (26.4)	0.00

Abbreviations: ACEIs, angiotensin-converting enzyme inhibitors; aDCSI, adapted diabetes complication severity index; ARBs, angiotensin receptor blockers; COPD, chronic obstructive pulmonary disease; DM, diabetes mellitus; DPP-4is, dipeptidylpeptidase-4 inhibitors; ER, emergency room; OHA, oral hypoglycemic agent; PDC, proportion of days covered; S.D., standardized difference; SUs, sulfonylureas; TZD, thiazolidinediones.

### Cardiovascular events, all-cause mortality, and hypoglycemia

In the main analysis of the as-treated approach, the median follow-up time for the composite major adverse cardiovascular events (MACEs) was 0.35, 0.46, and 0.52 years, in the basal insulin, TZD, and DPP-4i groups, respectively. There was no significant difference in the risk of MACEs, hypoglycemia, or individual cardiovascular (CV) events among the treatment groups (Table 3). In contrast, the hazard ratio (HR) of all-cause mortality was significantly lower in the TZD (HR 0.55, 95% CI 0.38–0.81) and DPP-4i (HR 0.56, 95% CI 0.39–0.82) groups than in the basal insulin group.

**Table 3:** Numbers of events, incidence rates and hazard ratios of the studied outcomes (MACEs, all-cause mortality, hypoglycemia and individual cardiovascular outcomes) (as-treated analysis).

	No. of events	Median (IQR) time to event (years)	Follow-up time (person-years)	Incidence rate (per 1000 person-years)	Hazard ratio
MACEs					
Basal insulin	36	0.40 (0.14–0.96)	4,035	8.92	Reference
TZDs	103	0.35 (0.16–0.90)	9,513	10.83	1.24 (0.85–1.82)
Basal insulin	37	0.38 (0.14–0.80)	3,984	9.29	Reference
DPP-4is	103	0.44 (0.19–0.95)	11,085	9.29	1.06 (0.73–1.54)
All-cause mortality					
Basal insulin	46	0.37 (0.25–0.73)	4,043	11.38	Reference
TZDs	61	0.57 (0.25–1.22)	9,543	6.39	**0.55 (0.38–0.81)**
Basal insulin	45	0.38 (0.25–0.73)	3,992	11.27	Reference
DPP-4is	69	0.52 (0.30–1.02)	11,140	6.19	**0.56 (0.39–0.82)**
Hypoglycemia					
Basal insulin	49	0.19 (0.08–0.72)	3,527	13.89	Reference
TZDs	117	0.42 (0.19–0.94)	8,532	13.71	1.01 (0.72–1.41)
Basal insulin	49	0.19 (0.09–0.72)	3,480	14.08	Reference
DPP-4is	101	0.37 (0.12–1.10)	10,091	10.01	0.78 (0.55–1.10)
Myocardial infarction					
Basal insulin	10	0.55 (0.16–1.16)	4,040	2.48	Reference
TZDs	27	0.38 (0.15–1.38)	9,536	2.83	1.16 (0.56–2.40)
Basal insulin	10	0.55 (0.16–1.16)	3,989	2.51	Reference
DPP-4is	20	0.37 (0.22–0.69)	11,131	1.80	0.75 (0.35–1.62)
Ischemic stroke					
Basal insulin	22	0.38 (0.13–1.12)	4,038	5.45	Reference
TZDs	68	0.33 (0.16–0.79)	9,520	7.14	1.35 (0.84–2.19)
Basal insulin	23	0.37 (0.13–1.12)	3,987	5.77	Reference
DPP-4is	73	0.45 (0.17–1.07)	11,098	6.58	1.21 (0.76–1.94)
Cardiovascular death					
Basal insulin	5	0.72 (0.21–0.76)	4,043	1.24	Reference
TZDs	9	0.39 (0.28–0.80)	9,543	0.94	0.77 (0.26–2.30)
Basal insulin	5	0.72 (0.21–0.76)	3,992	1.25	Reference
DPP-4is	13	0.38 (0.30–0.86)	11,140	1.17	1.03 (0.36–2.92)

Abbreviations: DPP-4is, dipeptidylpeptidase-4 inhibitors; IQR, interquartile range; MACEs, major adverse cardiovascular events; TZDs, thiazolidinediones.

### Sensitivity and subgroup analyses

The results of the sensitivity analyses are presented in Supplemental Tables S1–S4. With the intention-to-treat analytic approach, patients in the basal insulin, TZD, and DPP-4i groups were followed for 4.16, 4.52, and 4.09 years, respectively, and similar results were found for most of the outcomes, including MACEs and all-cause mortality. However, the risk of ischemic stroke was significantly higher in the TZD group (HR 1.25, 95% CI 1.03–1.50) than the basal insulin group. On the other hand, the risk of CV death was lower in both the TZD and DPP-4i treatment groups (HR 0.77 [95% CI 0.60–0.99] and 0.66 [0.51–0.86], respectively) than in the basal insulin group. In the remaining sensitivity analyses, the results were all consistent with the main analysis. For the comparison of basal insulin and TZDs with regard to all-cause mortality, the E-values were 3.04 for the HR point estimate and 1.77 for the lower limit of the HR confidence interval. It means that residual confounding could pull the observed association toward the null if there exists an unmeasured confounder having a relative risk association at least as large as 3.04 with both TZDs exposure and all-cause mortality. For the comparison of basal insulin and DPP-4is with regard to all-cause mortality, the E-values were 2.97 for the HR point estimate and 1.74 for the lower limit of the HR confidence interval.

**Figure 2:**
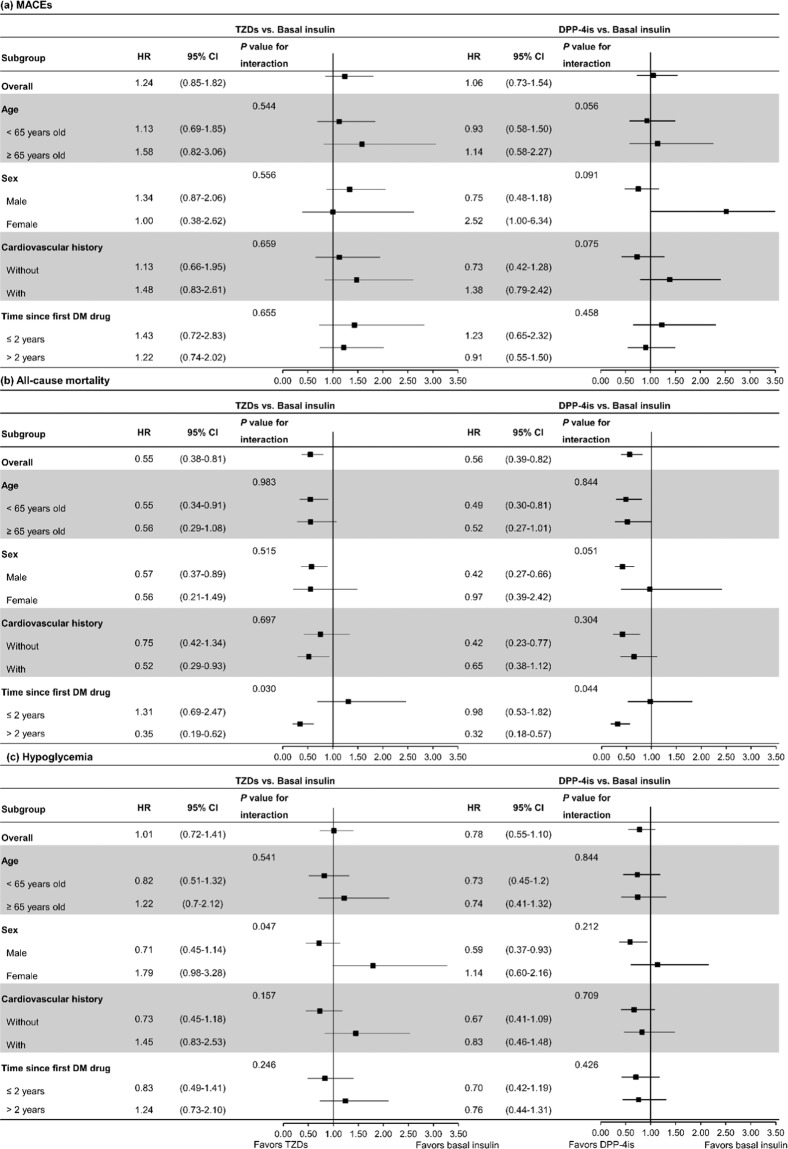
Subgroup analyses of (**a**) MACEs, (**b**) all-cause mortality, and (**c**) hypoglycemia. Abbreviations: CI, confidence interval; DM, diabetes mellitus; DPP-4is, dipeptidylpeptidase-4 inhibitors; HR, hazard ratio; MACEs, major adverse cardiovascular event; TZDs, thiazolidinediones.

For the subgroup analysis (Fig. 2), we found that the reduced risk of all-cause mortality associated with TZD/DPP-4i use was more prominent in patients whose diabetes had occurred for more than two years (TZD: HR 0.35 [95% CI 0.19–0.62]; DPP-4i: HR 0.32 [0.18–0.57]) (*P* value for interaction both <0.05). Moreover, females tended to have a higher risk of hypoglycemia than males when receiving TZD as the third antidiabetic agent (*P* value for interaction <0.05), although the increased risk of TZD compared to basal insulin in females was not significantly different (female HR 1.79 [95% CI 0.98–3.28] vs. male HR 0.71 [0.45–1.14]). No other subgroup differences were observed. The results of different types of basal insulins are presented in Supplemental Tables S5–S7, but the sample numbers were too small to conclude.

## Discussion

The present study showed that the risks of MACEs and hypoglycemia were similar in type 2 DM (T2DM) patients treated with basal insulin compared to TZDs and DPP-4is as an add-on to dual OHA combination therapy. However, patients treated with basal insulin presented a higher risk of all-cause mortality than patients treated with TZDs and DPP-4is, especially those who endured long-term diabetes. The consistent results in the sensitivity analyses, particularly the intention-to-treat (ITT) analysis with a much increased follow-up time, further validated these associations. These results indicate that TZDs or DPP-4is as add-on therapies after the failure of dual therapy could be a safer option than basal insulin as an add-on therapy, specifically for patients with more advanced DM.

The present study focused on comparing third antidiabetic agents because it has been revealed that the use of basal insulin is usually delayed; for instance, the results of a previous study found that Taiwanese patients received an average of 2.7 OHAs before starting basal insulin therapy^26^. Earlier studies on the treatment patterns of antidiabetic agents in Taiwan indicated that over 70% of dual therapy regimens involved a combination of metformin and sulfonylureas^14,15^. The resistance against the initiation of insulin may result from misconceptions about insulin, the inconvenience of injectable medications, and the easy accessibility of other oral treatment options, such as TZDs and DPP-4is^10^. While insulin has been demonstrated to preserve β-cells better than metformin and sulfonylureas by replacing endogenous insulin to induce β-cell rest^6–8^, similar effects were also found with TZDs and DPP-4is. TZDs are effective insulin sensitizers, and DPP-4is affect glucose regulation through multiple mechanisms by increasing incretin levels^12^. Both drugs not only preserve β-cells by reducing apoptosis but also improve β-cell function by maintaining or stimulating proliferation^27^. With similar pharmacological effects and uses in therapy, TZDs and DPP-4is are ideal comparators for evaluating the outcomes of basal insulin as a third-line therapy.

Several observational studies have explored the cardiovascular outcomes of insulin, but the findings have been inconsistent, especially among different active comparators^18–21,23^. When sulfonylureas were compared with insulin, the studies yielded comparable cardiovascular results between the treatments^18,19^. When DPP-4is were selected as a reference, the risk of macrovascular events appeared to be higher in patients receiving insulin treatment than in those receiving DPP-4i treatment. For instance, Nystrom *et al*. noted that the risk of fatal and non-fatal CVDs in the insulin arm was 1.39 times as much as in the DPP-4i arm for second-line treatment after metformin monotherapy^21^. Jil *et al*. also reported that the risk of composite outcome (non-fatal acute myocardial infarction (AMI), non-fatal stroke or all-cause death) was 1.6 times higher in patients receiving intensified treatment with insulin than in patients receiving intensified treatment with DPP-4is following dual-therapy failure^20^. However, both studies included short-acting insulin in the analyses, which might obscure the effect of basal insulin. In contrast, one study compared the cardiovascular risk of NPH insulin to DPP-4is as a third antidiabetic agent and showed no significant increased risk among the treatment groups^23^. However, no study has directly compared the cardiovascular events associated with insulin and those associated with TZDs. In one study that compared various glucose-lowering agents as add-on medications to metformin and used sulfonylurea as the reference group, the risks of CVDs in the TZD and DPP-4i groups were significantly lower, while the risk of CVDs in the basal insulin group was similar to that in the sulfonylurea reference group^19^.

Our findings showed similar risks of macrovascular events among the treatment groups, but the short exposure or follow-up times might have resulted in an insufficient effect or insignificant difference in the study drugs. In addition, our study population comprised relatively young patients with a short DM duration, and the reduced cardiovascular risk could be less pronounced in the young population. There remains debate as to whether intensive glycemic control leads to improved CV outcomes, no difference in risk or increased mortality. The inconsistent results in prior studies may originate from different study populations, the administration of early or late intensive glycemic control, and the lack of a long-term follow-up period in which cardiovascular benefits can be observed^2–5,24,28,29^.

Despite the inconsistent findings regarding CV events, nearly all the observational studies, including ours, found that insulin therapy was associated with an increased risk of all-cause mortality^18–21^. In addition, our study found that the increased risk of all-cause mortality associated with basal insulin therapy was even more prominent in patients who had more than two years of diabetic history. The results moderately corresponded with the ACCORD (Action to Control Cardiovascular Risk in Diabetes) trial^3^, which included an advanced DM population with an increased risk of mortality due to intensive glycemic treatment. The exact mechanism remains unclear; presumably, the increased risk could possibly be related to hypoglycemia and CV-related death. Hypoglycemia is known to provoke unstable hemodynamics and dysrhythmia and might result in an increased number of cardiovascular events and sudden death^30^. In the analysis by Kuo *et al*., after post-index hypoglycemia was controlled to account for the potential effect on all-cause mortality, the increased risks associated with insulin therapy no longer existed (the HR decreased from 1.48 [95% CI 1.01–2.17] to 1.30 [0.84–1.99])^22^. The latest American Diabetes Association guideline has recently revised the recommendations for choosing antidiabetic agents and suggests that a GLP-1 agonist, an injectable medication, is preferred over basal insulin to reduce the risk of hypoglycemia and weight gain^13^. While the present study showed that the risk of hypoglycemia was comparable among the treatment groups, the diagnosis of hypoglycemia may not be sensitive enough, and it is unlikely that the increased risks of all-cause mortality can all be attributed to the occurrence of hypoglycemia. More studies are warranted to investigate the mechanism of increased risk of death in patients treated with basal insulin.

Our study has several strengths. This study utilized a nationwide claims database, resulting in a large sample size to investigate the outcomes of basal insulin as a third antidiabetic agent for therapy intensification. In addition, financial issues do not affect the treatment choice or our study validity because the National Health Insurance in Taiwan did not impose specific reimbursement policy for the related use of TZD, DPP-4i, and basal insulin in diabetic patients. We also performed a series of sensitivity analyses to ascertain the robustness of the study results. The study results of MACEs, all-cause mortality, and hypoglycemia were consistent and therefore increased the credibility of our findings. Finally, this study provides additional insights into treatment strategies, revealing a safety concern associated with basal insulin, which is in line with the recommendation of the delayed use of basal insulin in the latest clinical guideline^13^.

There are several limitations in the present study. To ensure sufficient power in the outcome analysis, we utilized a broader definition for identifying patients with stable dual treatment (i.e., receiving metformin and one other OHA for more than 14 days in an outpatient setting) before starting the third antidiabetic agent. The definition might have resulted in enrolling patients who changed their treatment regimen frequently, but the results remained consistent in the sensitivity analysis, which only included patients who completed dual therapy for at least 90 days. This definition in this sensitivity analysis also aligned with the clinical guideline recommendation stating that a new antidiabetic agent should be added if an appropriate HbA1c level is not achieved after approximately three months of dual therapy^12^. Another limitation is the lack of laboratory results in the claims database. HbA1c is an important indicator of DM control. Although many proxies of disease severity (e.g., adapted Diabetes Complication Severity Index (aDCSI) score, time since first antihyperglycemic agents, disease burden, and HbA1c order rate) were adjusted to mitigate confounding effects, residual bias was still possible. Moreover, we failed to adjust for unmeasured confounders such as baseline body mass index (BMI), weight gain, and history of smoking in the analysis. Nonetheless, we reported the E-values, which indicate how strongly an unmeasured confounder must be related to the treatment and outcome to explain away a significant risk estimate. One of the strengths of the E-value approach is that it makes no assumptions specifying the nature of unmeasured confounder or the strength of the confounding association^31^. In our analysis of all-cause mortality, the E-values were respectively 3.04 and 2.97 for the comparison of basal insulin vs. TZDs and DPP-4is, and thus substantial unmeasured confounding would be needed to reduce the observed association to null. For example, several studies have assessed the impact of BMI and smoking on all-cause mortality^32–34^, and their risk estimates showed it might be implausible to have an unmeasured confounder associated with both treatment choice and all-cause mortality by a risk ratio of 3-fold. Last, similar to many other studies, we defined medication exposure only based on pharmacy dispensing records, and true medication adherence was unknown.

The present nationwide cohort study identified the safety outcomes of initiating basal insulin compared with TZDs and DPP-4is after the failure of dual therapy. No increased risk was found for cardiovascular events and hypoglycemia; however, an increased risk of all-cause mortality was observed in patients receiving basal insulin. Regarding safety concerns, as the latest clinical guideline suggested, there is no apparent benefit to initiating early basal insulin therapy when other OHA choices, such as DPP-4is and TZDs, are available. Further studies are needed to determine the mechanism of the increased risk in all-cause mortality in patients receiving basal insulin, especially those with advanced DM.

## Methods

### Data source

This retrospective cohort study utilized nationwide data from 2002–2015 from the National Health Insurance Research Database (NHIRD) in Taiwan. The claims cover more than 99.9% of the 23 million residents and contain complete records of outpatient, inpatient, ER, and pharmacy data. This study was approved by the Research Ethics Committee of the National Taiwan University Hospital (201710009RINC). Owing to the anonymous nature of the data, informed consent was waived by the ethics committee for the entire study. All methods were performed in accordance with the relevant guidelines and regulations.

### Study population

The study population consisted of T2DM patients aged between 20 and 80 years with at least two outpatient or one inpatient diagnosis of new-onset diabetes (International Classification of Diseases, Ninth Revision, Clinical Modification [ICD-9-CM] code 250) between January 1, 2003, and December 31, 2014. Patients who received metformin and one of the other OHAs (sulfonylureas, meglitinides, TZDs, DPP-4is, or α-glucosidase inhibitors) for at least 14 days in the outpatient setting preceding the administration of a third antidiabetic agent were identified. Patients who were administered basal insulin, TZDs, or DPP-4is as a third antidiabetic agent after 2008 were included, and the first date of triple therapy was defined as the index date. The included patients were required to continue the triple therapy for at least two prescription cycles. Patients were excluded from the analysis if two or more antidiabetic agents were added at the same time after dual therapy or if the third antidiabetic agent was not a DPP-4i, a TZD, or basal insulin.

### Exposure

The use of basal insulin (NPH, insulin glargine, and insulin detemir) as a third antidiabetic agent was compared to the use of TZDs (pioglitazone and rosiglitazone) and DPP-4is (sitagliptin, saxagliptin, vildagliptin, and linagliptin) separately. When comparing basal insulin and TZDs, we excluded patients with prior dual therapy containing TZDs. Likewise, when comparing basal insulin and DPP-4is, we excluded patients with prior dual therapy containing DPP-4is. The duration of each drug was calculated by days of drug supply, and when determining drug discontinuation, half of the supply days were considered a grace period to account for delayed refills^35^.

### Outcome definition

The study outcome included MACEs, all-cause mortality, hypoglycemia, and individual cardiovascular events. MACEs were defined as hospitalization with a primary diagnosis of AMI (ICD-9-CM codes 410.x), ischemic stroke (433.x, 434.x), or CV death. All-cause mortality was determined based on the National Death Registry, and CV deaths were further defined considering the following CV causes: (1) heart-related; (2) hypertension-related; (3) cerebrovascular-related; (4) artery-, arteriole-, or capillary-related; and (5) vein-related. Hypoglycemia was defined as an ER visit or hospitalization with a diagnosis of hypoglycemic coma (251.0), other hypoglycemia (251.1, 251.2), or diabetes with other specified manifestations (250.8 without co-diagnosis codes 259.8, 272.7, 681.x, 682.x, 689.x, 707.1–707.9, 709.3, 730.0–730.2, or 781.8)^36^. Patients were followed until the occurrence of the study outcome, start of a new antidiabetic agent, discontinuation of study drug (basal insulin, TZD or DPP-4i), death, or end of 2015, whichever came first. To identify all related events, MACEs, all-cause mortality, and individual CV outcomes were followed for an additional 30 days after starting a fourth antidiabetic agent or discontinuation of the study drug.

### Covariates

The study covariates, including age, sex, index year, clinical characteristics, and healthcare resource utilization (HCRU), were collected over a baseline period of one year prior to (and including) the index date. We included the following variables to assess and control for the baseline condition of diabetes: first antidiabetic agent, dual therapy medication other than metformin, time from the first antidiabetic agent to the index date (as a proxy of disease duration), duration of dual therapy, number of HbA1c tests in the past year, baseline proportion of days covered (PDC) by antidiabetic drugs (an estimate of medication adherence)^37^, aDCSI score (as a proxy of DM severity)^38^, and history of previous hypoglycemia. Patients’ HCRU in the past year, in terms of the numbers of outpatient visits, ER visits, and hospitalizations, were considered as proxies of their general health condition and disease burden.

The following comorbidities present during the baseline period were identified (Supplemental Table S8): myocardial infarction (MI), other coronary arterial diseases (CAD), peripheral vascular disease (PVD), cerebrovascular disease, heart failure (HF), dysrhythmia, valvular heart disease (VHD), hypertension, dyslipidemia, chronic kidney disease, diseases that possibly require the frequent use of corticosteroids (autoimmune diseases, transplantation, asthma or chronic obstructive pulmonary disease), and mental disorders (depression, bipolar disorder, schizophrenia or anxiety). The data were adjusted for comedications, including α-blockers, β-blockers, ACE inhibitors, ARBs, renin inhibitors, CCBs, diuretics, nitrates, digoxin, lipid-lowering agents, antiplatelet agents, anticoagulants, atypical antipsychotics, and systemic steroids.

### Statistical analysis

Patient demographics, baseline characteristics, diabetes condition, comorbidities, and comedications were compared between the users of basal insulin and TZDs or DPP-4is. Continuous variables were expressed as means ± standard deviations, and categorical variables were expressed as numbers and proportions. A PS model was developed by including all the covariates in the logistic regression^39^. The comparison groups were 1:2 matched by PS (using nearest-neighbor matching with a caliper width equal to 0.2 times the standard deviation of the logit of the PS)^40^ as well as a history of cardiovascular disease (i.e., presence of MI, CAD, PVD, cerebrovascular disease, HF, dysrhythmia, or VHD). The balance in the baseline covariates between the exposure groups before and after PS matching was evaluated by standardized differences. A standardized difference of less than 0.1 indicated a negligible difference^41^. Cox proportional hazards models were then used to estimate the HRs of each study outcome between the comparison groups of basal insulin and TZDs/DPP-4is.

A series of sensitivity analyses were performed to ascertain the consistency of the results. First, an ITT approach was applied for all study outcomes except hypoglycemia. In the ITT analysis, every patient was followed until the occurrence of the study outcome, death, or the end of 2015. Second, the dual therapy before the intensification therapy was confined to the combination of metformin and sulfonylureas. Third, the definition of dual therapy was restricted to combined use for at least 90 days to obtain results for a population in which the treatment strategy was in accordance with the clinical guideline^42^. Fourth, a strict definition of hypoglycemia was used to include only the events recorded in the primary diagnosis. Fifth, to study the hypoglycemia outcome, one additional censoring criterion was applied; patient follow-up was censored when any of the first two antidiabetic agents was discontinued since the risk of hypoglycemia could change after altering the triple combination. Last, to evaluate the robustness of significant associations to potential unmeasured confounders, the E-values were calculated to present the minimum strength of the association that an unmeasured confounder would need to have with both the treatment and outcome to explain away the observed treatment-outcome association^31^.

Subgroup analyses were conducted to evaluate the differential effects of exposure among subpopulations with regard to (1) age (<65, ≥65 years); (2) sex (male, female); (3) history of cardiovascular disease (without, with); and (4) diabetes duration (<2, ≥2 years). Due to the limited number of patients with previous hypoglycemic events, we did not stratify the subgroups according to the presence or absence of hypoglycemia history. To explore the clinical outcomes of different types of basal insulins, we further divided basal insulins into NPH, insulin glargine, and insulin detemir.

All data analyses were performed using SAS, version 9.4 (SAS Institute, Cary, NC, USA). Two-sided *P* values less than 0.05 were considered statistically significant.

## Supplementary information

Supplementary Information.

## Data Availability

The data that support the findings of this study are available from Taiwan’s Ministry of Health and Welfare but restrictions apply to the availability of these data, which were used under license for the current study, and so are not publicly available.

## References

[1] American Diabetes A (2018). 6. Glycemic Targets: Standards of Medical Care in Diabetes-2018. Diabetes Care.

[2] Holman RR, Paul SK, Bethel MA, Matthews DR, Neil HA (2008). 10-year follow-up of intensive glucose control in type 2 diabetes. N. Engl. J. Med..

[3] Gerstein HC (2008). Effects of intensive glucose lowering in type 2 diabetes. N. Engl. J. Med..

[4] Patel A (2008). Intensive blood glucose control and vascular outcomes in patients with type 2 diabetes. N. Engl. J. Med..

[5] Duckworth W (2009). Glucose control and vascular complications in veterans with type 2 diabetes. N. Engl. J. Med..

[6] Chen HS (2008). Beneficial effects of insulin on glycemic control and beta-cell function in newly diagnosed type 2 diabetes with severe hyperglycemia after short-term intensive insulin therapy. Diabetes Care.

[7] Weng J (2008). Effect of intensive insulin therapy on beta-cell function and glycaemic control in patients with newly diagnosed type 2 diabetes: a multicentre randomised parallel-group trial. Lancet.

[8] Pistrosch F (2013). Effects of insulin glargine versus metformin on glycemic variability, microvascular and beta-cell function in early type 2 diabetes. Acta Diabetol..

[9] Rosenstock J (2005). Reduced hypoglycemia risk with insulin glargine: a meta-analysis comparing insulin glargine with human NPH insulin in type 2 diabetes. Diabetes Care.

[10] Karter AJ (2010). Barriers to insulin initiation: the translating research into action for diabetes insulin starts project. Diabetes care.

[11] American Diabetes A (2017). 8. Pharmacologic Approaches to Glycemic Treatment: Standards of Medical Care in Diabetes-2017. Diabetes Care.

[12] American Diabetes A (2018). 8. Pharmacologic Approaches to Glycemic Treatment: Standards of Medical Care in Diabetes-2018. Diabetes Care.

[13] American Diabetes A (2019). 9. Pharmacologic Approaches to Glycemic Treatment: Standards of Medical Care in Diabetes-2019. Diabetes Care.

[14] Chang CH, Jiang YD, Chung CH, Ho LT, Chuang LM (2012). National trends in anti-diabetic treatment in Taiwan, 2000-2009. J. Formos. Med. Assoc..

[15] Chu WM (2017). The prescribing trend of oral antidiabetic agents for type 2 diabetes in Taiwan: An 8-year population-based study. Medicine.

[16] Datta-Nemdharry P, Thomson A, Beynon J, Donegan K (2017). Patterns of anti-diabetic medication use in patients with type 2 diabetes mellitus in England and Wales. Pharmacoepidemiol. Drug. Saf..

[17] Khunti K (2018). Patterns of glycaemic control in patients with type 2 diabetes mellitus initiating second-line therapy after metformin monotherapy: Retrospective data for 10 256 individuals from the United Kingdom and Germany. Diabetes Obes. Metab..

[18] Roumie CL (2014). Association between intensification of metformin treatment with insulin vs sulfonylureas and cardiovascular events and all-cause mortality among patients with diabetes. JAMA.

[19] Ekstrom N (2016). Cardiovascular safety of glucose-lowering agents as add-on medication to metformin treatment in type 2 diabetes: report from the Swedish National Diabetes Register. Diabetes Obes. Metab..

[20] Jil M, Rajnikant M, Richard D, Iskandar I (2017). The effects of dual-therapy intensification with insulin or dipeptidylpeptidase-4 inhibitor on cardiovascular events and all-cause mortality in patients with type 2 diabetes: A retrospective cohort study. Diab Vasc. Dis. Res..

[21] Nystrom T (2017). Second line initiation of insulin compared with DPP-4 inhibitors after metformin monotherapy is associated with increased risk of all-cause mortality, cardiovascular events, and severe hypoglycemia. Diabetes Res. Clin. Pract..

[22] Kuo, S., Yang, C. T., Wu, J. S. & Ou, H. T. Effects on clinical outcomes of intensifying triple oral antidiabetic drug (OAD) therapy by initiating insulin versus enhancing OAD therapy in patients with type 2 diabetes: A nationwide population-based, propensity-score-matched cohort study. *Diabetes Obes Metab*, 10.1111/dom.13525 (2018). 10.1111/dom.13525 PMC6329671 30187666

[23] Moura CS (2018). Treatment Discontinuation and Clinical Events in Type 2 Diabetes Patients Treated with Dipeptidyl Peptidase-4 Inhibitors or NPH Insulin as Third-Line Therapy. J. Diabetes Res..

[24] Gerstein HC (2012). Basal insulin and cardiovascular and other outcomes in dysglycemia. N. Engl. J. Med..

[25] *WHO Collaborating Centre for Drug Statistics Methodology, ATC classification index with DDDs, 2020. Oslo, Norway 2019*, https://www.whocc.no/atc_ddd_index/ (2020).

[26] Lin SD (2015). Glycosylated hemoglobin level and number of oral antidiabetic drugs predict whether or not glycemic target is achieved in insulin-requiring type 2 diabetes. Prim. Care Diabetes.

[27] Wajchenberg B (2007). L. beta-cell failure in diabetes and preservation by clinical treatment. Endocr. Rev..

[28] Intensive blood-glucose control with sulphonylureas or insulin compared with conventional treatment and risk of complications in patients with type 2 diabetes (UKPDS 33). UK Prospective Diabetes Study (UKPDS) Group. *Lancet* **352**, 837–853 (1998). 9742976

[29] Skyler JS (2009). Intensive glycemic control and the prevention of cardiovascular events: implications of the ACCORD, ADVANCE, and VA Diabetes Trials: a position statement of the American Diabetes Association and a Scientific Statement of the American College of Cardiology Foundation and the American Heart Association. J. Am. Coll. Cardiol..

[30] Frier BM, Schernthaner G, Heller SR (2011). Hypoglycemia and cardiovascular risks. Diabetes care.

[31] VanderWeele TJ, Ding P (2017). Sensitivity Analysis in Observational Research: Introducing the E-Value. Ann. Intern. Med..

[32] Aune D (2016). BMI and all cause mortality: systematic review and non-linear dose-response meta-analysis of 230 cohort studies with 3.74 million deaths among 30.3 million participants. BMJ.

[33] Winter JE, MacInnis RJ, Nowson CA (2017). The Influence of Age the BMI and All-Cause Mortality Association: A Meta-Analysis. J. Nutr. Health Aging.

[34] Lariscy JT, Hummer RA, Rogers RG (2018). Cigarette Smoking and All-Cause and Cause-Specific Adult Mortality in the United States. Demography.

[35] Lund JL, Richardson DB, Stürmer T (2015). The active comparator, new user study design in pharmacoepidemiology: historical foundations and contemporary application. Curr. Epidemiol. Rep..

[36] Ginde AA, Blanc PG, Lieberman RM, Camargo CA (2008). Validation of ICD-9-CM coding algorithm for improved identification of hypoglycemia visits. BMC Endocr. Disord..

[37] Martin BC (2009). Contrasting measures of adherence with simple drug use, medication switching, and therapeutic duplication. Ann. Pharmacother..

[38] Chang HY, Weiner JP, Richards TM, Bleich SN, Segal JB (2012). Validating the adapted Diabetes Complications Severity Index in claims data. Am. J. Manag. Care.

[39] Dehejia RH, Wahba S (2002). Propensity Score-Matching Methods for Nonexperimental Causal Studies. Rev. Econ. Stat..

[40] Austin PC (2011). Optimal caliper widths for propensity-score matching when estimating differences in means and differences in proportions in observational studies. Pharm. Stat..

[41] Austin PC (2009). Using the Standardized Difference to Compare the Prevalence of a Binary Variable Between Two Groups in Observational Research. Commun. Stat-Simul C..

[42] American Diabetes A (2012). Standards of medical care in diabetes-2012. Diabetes care.

