# Supplementary material for: Clinical outcomes of basal insulin and oral antidiabetic agents as an add-on to dual therapy in patients with type 2 diabetes mellitus
Source: Sci Rep. 2020 Apr 1;10:5746. doi: 10.1038/s41598-020-62646-z (PMC7113251; doi:10.1038/s41598-020-62646-z)
Supplement: Supplementary file 1 — Supplementary Information. [file 41598_2020_62646_MOESM1_ESM.docx]

**SUPPLEMENTAL INFORMATION**

**ARTICLE TITLE:** Clinical outcomes of basal insulin and oral antidiabetic agents as an add-on to dual therapy in patients with type 2 diabetes mellitus

**AUTHORS:** Chih-Ning Cheng, Chih-Yuan Wang, Hung-Wei Lin, Ting-Yu Chang, Hsu-Ju Lin, Chiahung Chou, Fang-Ju Lin

**Supplemental Table S1.** Sensitivity analyses of the numbers of events, incidence rates and hazard ratios of MACE, all-cause mortality, and individual cardiovascular outcomes (intention-to-treat analysis)

|  | No. of events | Median (IQR) time to events years | Follow-up time (person-years) | Incidence rate (per 1000 person-years) | Hazard ratio |
| --- | --- | --- | --- | --- | --- |
| MACEs |  | | | | |
| Basal insulin | 321 | 2.77 (1.24-3.99) | 24,875 | 12.90 | Reference |
| TZDs | 647 | 2.45 (1.11-4.27) | 50,444 | 12.83 | 0.99 (0.87-1.13) |
| Basal insulin | 322 | 2.65 (1.24-3.98) | 24,613 | 13.08 | Reference |
| DPP-4is | 577 | 2.32 (1.13-3.63) | 46,912 | 12.30 | 0.93 (0.81-1.07) |
| All-cause mortality |  | | | | |
| Basal insulin | 631 | 2.40 (1.16-4.12) | 25,419 | 24.82 | Reference |
| TZDs | 913 | 2.87 (1.46-4.50) | 51,713 | 17.65 | **0.71 (0.64-0.78)** |
| Basal insulin | 628 | 2.35 (1.17-3.99) | 25,156 | 24.96 | Reference |
| DPP-4is | 798 | 2.55 (1.33-3.92) | 47,910 | 16.66 | **0.67 (0.61-0.75)** |
| Myocardial infarction |  | | | | |
| Basal insulin | 92 | 3.03 (1.61-4.63) | 25,238 | 3.65 | Reference |
| TZDs | 155 | 2.58 (1.16-4.48) | 51,357 | 3.02 | 0.82 (0.63-1.06) |
| Basal insulin | 92 | 2.84 (1.36-4.23) | 24,973 | 3.68 | Reference |
| DPP-4is | 142 | 2.78 (1.53-3.83) | 47,646 | 2.98 | 0.79 (0.61-1.03) |
| Ischemic stroke |  | | | | |
| Basal insulin | 153 | 2.38 (0.99-3.92) | 25,048 | 6.11 | Reference |
| TZDs | 387 | 2.39 (1.02-3.98) | 50,781 | 7.62 | **1.25 (1.03-1.50)** |
| Basal insulin | 154 | 2.39 (1.12-3.93) | 24,786 | 6.21 | Reference |
| DPP-4is | 353 | 2.23 (1.01-3.54) | 47,163 | 7.48 | 1.19 (0.98-1.43) |
| Cardiovascular death |  | | | | |
| Basal insulin | 104 | 3.18 (1.41-4.45) | 25,419 | 4.09 | Reference |
| TZDs | 165 | 3.01 (1.63-4.70) | 51,713 | 3.19 | **0.77 (0.60-0.99)** |
| Basal insulin | 103 | 3.18 (1.42-4.40) | 25,156 | 4.09 | Reference |
| DPP-4is | 127 | 2.65 (1.33-4.14) | 47,910 | 2.65 | **0.66 (0.51-0.86)** |

Abbreviations: DPP-4is, dipeptidylpeptidase-4 inhibitors; MACEs, major adverse cardiovascular events; TZDs, thiazolidinediones.

**Supplemental Table S2.** Sensitivity analyses of the numbers of events, incidence rates and hazard ratios of MACE, all-cause mortality, and hypoglycemia (with dual therapy consisting of metformin and sulfonylurea)

|  | No. of events | Median (IQR) time to events years | Follow-up time (person-years) | Incidence rate (per 1000 person-years) | Hazard ratio |
| --- | --- | --- | --- | --- | --- |
| MACEs |  | | | | |
| Basal insulin | 33 | 0.42 (0.15-1.12) | 3,771 | 8.75 | Reference |
| TZDs | 89 | 0.38 (0.17-0.85) | 8,893 | 10.01 | 1.16 (0.78-1.73) |
| Basal insulin | 34 | 0.40 (0.15-1.12) | 3,816 | 8.91 | Reference |
| DPP-4is | 101 | 0.51 (0.20-0.96) | 10,698 | 9.44 | 1.11 (0.75-1.64) |
| All-cause mortality |  | | | | |
| Basal insulin | 44 | 0.38 (0.25-0.75) | 3,779 | 11.64 | Reference |
| TZDs | 65 | 0.80 (0.27-1.36) | 8,917 | 7.29 | **0.61 (0.42-0.90)** |
| Basal insulin | 44 | 0.38 (0.25-0.75) | 3,824 | 11.51 | Reference |
| DPP-4is | 76 | 0.60 (0.35-1.05) | 10,742 | 7.08 | **0.62 (0.43-0.90)** |
| Hypoglycemia |  | | | | |
| Basal insulin | 49 | 0.19 (0.08-0.72) | 3,299 | 14.85 | Reference |
| TZDs | 104 | 0.41 (0.19-0.92) | 7,963 | 13.06 | 0.90 (0.64-1.26) |
| Basal insulin | 47 | 0.19 (0.08-0.90) | 3,337 | 14.08 | Reference |
| DPP-4is | 102 | 0.39 (0.12-1.00) | 9,767 | 10.44 | 0.81 (0.58-1.15) |

Abbreviations: DPP-4i, dipeptidylpeptidase-4 inhibitors; MACEs, major adverse cardiovascular events; TZD, thiazolidinediones.

**Supplemental Table S3.** Sensitivity analyses of the numbers of events, incidence rates and hazard ratios of MACE, all-cause mortality, and hypoglycemia (with at least 90 days of dual therapy)

|  | No. of events | Median (IQR) time to events years | Follow-up time (person-years) | Incidence rate (per 1000 person-years) | Hazard ratio |
| --- | --- | --- | --- | --- | --- |
| MACEs |  | | | | |
| Basal insulin | 25 | 0.44 (0.21-1.16) | 2,544 | 9.83 | Reference |
| TZDs | 61 | 0.38 (0.22-0.88) | 5,816 | 10.49 | 1.06 (0.67-1.69) |
| Basal insulin | 25 | 0.44 (0.21-1.16) | 2,529 | 9.89 | Reference |
| DPP-4is | 49 | 0.72 (0.17-1.95) | 6,986 | 7.01 | 0.73 (0.45-1.18) |
| All-cause mortality |  | | | | |
| Basal insulin | 29 | 0.38 (0.25-0.82) | 2,549 | 11.38 | Reference |
| TZDs | 36 | 0.40 (0.23-1.15) | 5,829 | 6.18 | **0.54 (0.33-0.88)** |
| Basal insulin | 29 | 0.38 (0.25-0.82) | 2,534 | 11.44 | Reference |
| DPP-4is | 31 | 0.85 (0.39-1.33) | 7,008 | 4.42 | **0.38 (0.23-0.64)** |
| Hypoglycemia |  | | | | |
| Basal insulin | 27 | 0.30 (0.10-1.09) | 2,261 | 11.94 | Reference |
| TZDs | 75 | 0.39 (0.19-0.90) | 5,234 | 14.33 | 1.21 (0.78-1.88) |
| Basal insulin | 26 | 0.37 (0.11-1.09) | 2,249 | 11.56 | Reference |
| DPP-4is | 58 | 0.46 (0.16-1.00) | 6,433 | 9.02 | 0.84 (0.53-1.34) |

Abbreviations: DPP-4i, dipeptidylpeptidase-4 inhibitors; MACEs, major adverse cardiovascular events; TZD, thiazolidinediones.

**Supplemental Table S4.** Sensitivity analyses of the numbers of events, incidence rates and hazard ratios of hypoglycemia (additional criterion for censoring and outcome definitions based on only the primary diagnosis)

|  | No. of events | Median (IQR) time to events years | Follow-up time (person-years) | Incidence rate (per 1000 person-years) | Hazard ratio |
| --- | --- | --- | --- | --- | --- |
| Hypoglycemia – additional criterion for censoring | | | | | |
| Basal insulin | 45 | 0.18 (0.08-0.47) | 3,091 | 14.56 | Reference |
| TZDs | 112 | 0.41 (0.19-0.94) | 7,920 | 14.14 | 1.00 (0.71-1.42) |
| Basal insulin | 45 | 0.19 (0.08-0.47) | 3,050 | 14.75 | Reference |
| DPP-4is | 91 | 0.35 (0.12-1.05) | 8,813 | 10.33 | 0.77 (0.54-1.10) |
| Hypoglycemia – only based on primary diagnosis | | | | | |
| Basal insulin | 43 | 0.20 (0.08-0.93) | 3,534 | 12.17 | Reference |
| TZDs | 93 | 0.43 (0.19-0.79) | 8,538 | 10.89 | 0.91 (0.64-1.31) |
| Basal insulin | 41 | 0.30 (0.09-0.93) | 3,486 | 11.76 | Reference |
| DPP-4is | 81 | 0.37 (0.12-1.10) | 10,104 | 8.02 | 0.74 (0.51-1.08) |

Abbreviations: DPP-4i, dipeptidylpeptidase-4 inhibitors; TZD, thiazolidinediones.

**Supplemental Table S5.** Numbers of events, incidence rates and hazard ratios of MACE between different basal insulins (NPH, insulin glargine, and insulin detemir) and TZD / DPP-4is matched patients

|  | No. of patients | No. of events | Median (IQR) time to event (years) | Follow-up time (person-years) | Incidence rate (per 1000 person-years) | Hazard ratio |
| --- | --- | --- | --- | --- | --- | --- |
| Basal insulin | 6,101 | 36 | 0.40 (0.14-0.96) | 4,035 | 8.92 | Reference |
| TZDs | 11,823 | 103 | 0.35 (0.16-0.90) | 9,513 | 10.83 | 1.24 (0.85-1.82) |
| Basal insulin | 6,051 | 37 | 0.38 (0.14-0.80) | 3,984 | 9.29 | Reference |
| DPP-4is | 11,900 | 103 | 0.44 (0.19-0.95) | 11,085 | 9.29 | 1.06 (0.73-1.54) |
| NPH | 607 | -**^†^** | -**^†^** | 310 | 6.44 | Reference |
| TZDs | 1,199 | 10 | 0.39 (0.19-0.80) | 1,021 | 9.80 | 1.49 (0.32-6.86) |
| NPH | 608 | -**^†^** | -**^†^** | 310 | 6.45 | Reference |
| DPP-4is | 1,185 | 11 | 0.54 (0.31-0.85) | 1,146 | 9.60 | 1.28 (0.29-5.80) |
| Insulin glargine | 3,459 | 16 | 0.35 (0.13-0.96) | 2,354 | 6.80 | Reference |
| TZDs | 6,689 | 69 | 0.44 (0.22-1.16) | 5,357 | 12.88 | **1.91 (1.11-3.30)** |
| Insulin glargine | 3,401 | 17 | 0.36 (0.15-0.80) | 2,316 | 7.34 | Reference |
| DPP-4is | 6,700 | 61 | 0.38 (0.17-0.75) | 6,232 | 9.79 | 1.43 (0.83-2.45) |
| Insulin detemir | 2,035 | 18 | 0.39 (0.13-0.76) | 1,372 | 13.12 | Reference |
| TZDs | 3,935 | 24 | 0.19 (0.10-0.36) | 3,137 | 7.65 | 0.62 (0.34-1.14) |
| Insulin detemir | 2,042 | 18 | 0.39 (0.13-0.76) | 1,358 | 13.26 | Reference |
| DPP-4is | 4,015 | 31 | 0.44 (0.20-1.48) | 3,709 | 8.36 | 0.66 (0.37-1.19) |

**^†^**To protect the anonymity of patients, data with a very small sample size were not allowed to provide.

Abbreviations: DPP-4i, dipeptidylpeptidase-4 inhibitors; IQR, Interquartile range; MACEs, major adverse cardiovascular events; TZD, thiazolidinediones.

**Supplemental Table S6.** Numbers of events, incidence rates and hazard ratios of all-cause mortality between different basal insulins (NPH, insulin glargine, and insulin detemir) and TZD / DPP-4is matched patients

|  | No. of patients | No. of events | Median (IQR) time to event (years) | Follow-up time (person-years) | Incidence rate (per 1000 person-years) | Hazard ratio |
| --- | --- | --- | --- | --- | --- | --- |
| Basal insulin | 6,101 | 46 | 0.37 (0.25-0.73) | 4,043 | 11.38 | Reference |
| TZDs | 11,823 | 61 | 0.57 (0.25-1.22) | 9,543 | 6.39 | **0.55 (0.38-0.81)** |
| Basal insulin | 6,051 | 45 | 0.38 (0.25-0.73) | 3,992 | 11.27 | Reference |
| DPP-4is | 11,900 | 69 | 0.52 (0.30-1.02) | 11,140 | 6.19 | **0.56 (0.39-0.82)** |
| NPH | 607 | 9 | 0.35 (0.30-0.69) | 310 | 28.99 | Reference |
| TZDs | 1,199 | 7 | 1.21 (0.57-1.45) | 1,022 | 6.85 | **0.20 (0.07-0.52)** |
| NPH | 608 | 9 | 0.35 (0.30-0.69) | 310 | 29.00 | Reference |
| DPP-4is | 1,185 | 6 | -**^†^** | 1,151 | 5.21 | **0.16 (0.06-0.45)** |
| Insulin glargine | 3,459 | 20 | 0.37 (0.23-0.68) | 2,357 | 8.49 | Reference |
| TZDs | 6,689 | 37 | 0.57 (0.24-1.22) | 5,377 | 6.88 | 0.80 (0.46-1.38) |
| Insulin glargine | 3,401 | 20 | 0.37 (0.23-0.68) | 2,319 | 8.62 | Reference |
| DPP-4is | 6,700 | 39 | 0.62 (0.34-1.33) | 6,261 | 6.23 | 0.72 (0.42-1.24) |
| Insulin detemir | 2,035 | 17 | 0.39 (0.25-0.76) | 1,377 | 12.35 | Reference |
| TZDs | 3,935 | 17 | 0.43 (0.28-0.80) | 3,145 | 5.41 | **0.44 (0.22-0.86)** |
| Insulin detemir | 2,042 | 16 | 0.48 (0.25-0.77) | 1,363 | 11.74 | Reference |
| DPP-4is | 4,015 | 24 | 0.46 (0.24-0.78) | 3,728 | 6.44 | 0.61 (0.32-1.16) |

**^†^**To protect the anonymity of patients, data with a very small sample size were not allowed to provide.

Abbreviations: DPP-4i, dipeptidylpeptidase-4 inhibitors; IQR, Interquartile range; TZD, thiazolidinediones.

**Supplemental Table S7.** Numbers of events, incidence rates and hazard ratios of hypoglycemia between different basal insulins (NPH, insulin glargine, and insulin detemir) and TZD / DPP-4is matched patients

|  | No. of patients | No. of events | Median (IQR) time to event (years) | Follow-up time (person-years) | Incidence rate (per 1000 person-years) | Hazard ratio |
| --- | --- | --- | --- | --- | --- | --- |
| Basal insulin | 6,101 | 49 | 0.19 (0.08-0.72) | 3,527 | 13.89 | Reference |
| TZDs | 11,823 | 117 | 0.42 (0.19-0.94) | 8,532 | 13.71 | 1.01 (0.72-1.41) |
| Basal insulin | 6,051 | 49 | 0.19 (0.09-0.72) | 3,480 | 14.08 | Reference |
| DPP-4is | 11,900 | 101 | 0.37 (0.12-1.10) | 10,091 | 10.01 | 0.78 (0.55-1.10) |
| NPH | 607 | -**^†^** | -**^†^** | 261 | 3.83 | Reference |
| TZDs | 1,199 | 7 | 0.43 (0.39-0.62) | 919 | 7.62 | 1.69 (0.21-13.71) |
| NPH | 608 | -**^†^** | -**^†^** | 261 | 3.83 | Reference |
| DPP-4is | 1,185 | 5 | -**^†^** | 1,051 | 4.76 | 1.38 (0.16-12.04) |
| Insulin glargine | 3,459 | 33 | 0.19 (0.08-0.47) | 2,061 | 16.01 | Reference |
| TZDs | 6,689 | 66 | 0.34 (0.19-0.94) | 4,807 | 13.73 | 0.87 (0.57-1.33) |
| Insulin glargine | 3,401 | 33 | 0.19 (0.09-0.47) | 2,028 | 16.27 | Reference |
| DPP-4is | 6,700 | 56 | 0.38 (0.14-1.42) | 5,676 | 9.87 | 0.65 (0.42-1.01) |
| Insulin detemir | 2,035 | 15 | 0.20 (0.04-0.93) | 1,205 | 12.44 | Reference |
| TZDs | 3,935 | 44 | 0.51 (0.13-1.06) | 2,804 | 15.69 | 1.29 (0.72-2.32) |
| Insulin detemir | 2,042 | 15 | 0.20 (0.04-0.93) | 1,191 | 12.59 | Reference |
| DPP-4is | 4,015 | 40 | 0.32 (0.09-0.87) | 3,366 | 11.88 | 1.06 (0.58-1.93) |

**^†^**To protect the anonymity of patients, data with a very small sample size were not allowed to provide.

Abbreviations: DPP-4i, dipeptidylpeptidase-4 inhibitors; IQR, Interquartile range; TZD, thiazolidinediones.

**Supplemental Table S8.** International Classification of Diseases, 9^th^ edition, Clinical Modification (ICD-9-CM) codes used to define the baseline covariates

| Complications | ICD-9-CM diagnosis codes |
| --- | --- |
| Myocardial infarction | 410, 412 |
| Other coronary arterial disease | 413, 414, 429.2 |
| Cerebrovascular disease | 430-438 |
| Hypertension | 401-405 |
| Dyslipidemia | 272 |
| Heart failure | 398.91, 402.01, 402.11, 402.91, 404.01, 404.03, 404.11, 404.13, 404.91, 404.93, 425.4-425.9, 428.x |
| Peripheral vascular disease | 440.x, 441.2, 441.4, 441.7, 441.9, 443.1-443.9, 447.1, 557.1, 557.9, V43.4 |
| Dysrhythmia | 427 |
| Valvular heart disease | 093.2, 394.x–397.x, 424.x, 746.3–746.6, V42.2, V43.3 |
| Depression | 296.2, 296.3, 300.4, 311 |
| Bipolar disorder | 296.0, 296.1, 296.4-296.8 |
| Schizophrenia | 295 |
| Anxiety | 300, 301, 309.21 |
| Chronic kidney disease | 403.11, 403.91, 404.12, 404.13, 404.92, 404.93, 585, 586, 588.0, V42.0, V45.1, V56.x |
| Malignancy | 140-208 |
| Autoimmune disease | 99.3, 135, 136.1, 242, 245.2, 255.4, 281.0, 283.0, 287.3, 335.20, 340, 358.0, 390-398, 446 (except 446.3, 446.6), 555, 556, 571.49, 571.6, 579.0, 694.4, 695.4, 696.0, 696.1, 696.8, 710.0-710.4, 711.1, 714, 720.0, 725 |
| Transplantation | V42 |
| Asthma/Chronic obstructive pulmonary disease | 491, 492, 493, 496 |
